# Are School Absences Correlated with Influenza Surveillance Data in England? Results from Decipher My Data—A Research Project Conducted through Scientific Engagement with Schools

**DOI:** 10.1371/journal.pone.0146964

**Published:** 2016-03-02

**Authors:** Robert W. Aldridge, Andrew C. Hayward, Nigel Field, Charlotte Warren-Gash, Colette Smith, Richard Pebody, Declan Fleming, Shane McCracken, Decipher my Data project and schools

**Affiliations:** 1 Institute of Health Informatics, University College London, 222 Euston Road, London, NW1 2DA, United Kingdom; 2 The Farr Institute of Health Informatics Research, University College London, 222 Euston Road, London, NW1 2DA, United Kingdom; 3 Research Department of Infection and Population Health, University College London, London, United Kingdom; 4 Centre for Infectious Disease Surveillance and Control, Public Health England, London, United Kingdom; 5 Pate’s Grammar School, Cheltenham, United Kingdom; 6 Gallomanor Communications Ltd, Bath, United Kingdom; University of Massachusetts, UNITED STATES

## Abstract

**Background:**

School aged children are a key link in the transmission of influenza. Most cases have little or no interaction with health services and are therefore missed by the majority of existing surveillance systems. As part of a public engagement with science project, this study aimed to establish a web-based system for the collection of routine school absence data and determine if school absence prevalence was correlated with established surveillance measures for circulating influenza.

**Methods:**

We collected data for two influenza seasons (2011/12 and 2012/13). The primary outcome was daily school absence prevalence (weighted to make it nationally representative) for children aged 11 to 16. School absence prevalence was triangulated graphically and through univariable linear regression to Royal College of General Practitioners (RCGP) influenza like illness (ILI) episode incidence rate, national microbiological surveillance data on the proportion of samples positive for influenza (A+B) and with Rhinovirus, RSV and laboratory confirmed cases of Norovirus.

**Results:**

27 schools submitted data over two respiratory seasons. During the first season, levels of influenza measured by school absence prevalence and established surveillance were low. In the 2012/13 season, a peak of school absence prevalence occurred in week 51, and week 1 in RCGP ILI surveillance data. Linear regression showed a strong association between the school absence prevalence and RCGP ILI (All ages, and 5–14 year olds), laboratory confirmed cases of influenza A & B, and weak evidence for a linear association with Rhinovirus and Norovirus.

**Interpretation:**

This study provides initial evidence for using routine school illness absence prevalence as a novel tool for influenza surveillance. The network of web-based data collection platforms we established through active engagement provides an innovative model of conducting scientific research and could be used for a wide range of infectious disease studies in the future.

## Introduction

School-aged children are an important group in the transmission of influenza with well documented outbreaks of influenza and influenza like illness (ILI) occurring in schools.[1] Studies examining the community burden of seasonal and pandemic influenza have demonstrated that children have significantly higher rates of PCR-confirmed disease and serological infection with influenza A than adults.[2]

Many people with influenza do not see a doctor as it is often a mild self-limiting illness, however, many of the surveillance systems use data collected from patients’ interactions with the health service.[2] Current surveillance systems may therefore be less sensitive to milder forms of the infection and underestimate the amount of influenza transmission within the population, particularly in the early stages of an epidemic.

Boarding schools taking part in the Medical Officers of Schools Association scheme send reports of various illnesses, including ILI and swabbing to Public Health England (PHE) for surveillance purposes during school term. The system can potentially provide an early indication of the influenza strains for the forthcoming season and has also been used to measure the impact of influenza vaccination, though by their nature, these existing surveillance data in schools will not be representative of most children in England.[3]

Pilot studies using school absence data for influenza surveillance have been conducted previously in England and demonstrated the potential usefulness of such an approach. The majority of data from these studies was collected in primary schools, with three secondary schools providing data for one influenza season.[4,5] These studies relied on historic electronic records from schools or daily upload of absence data, which can be administratively burdensome with minimal benefit to schools providing this information. We sought to address these issues by receiving data from schools across England on a weekly basis and involving students in the collection, submission and analysis of data for educational purposes.

This study aimed to establish an electronic system for the collection of routine school absence data as part of a Public Engagement with Science project and to determine if school absence prevalence was correlated with established healthcare surveillance measures for circulating influenza.

## Methods

Schools that had taken part in a previous scientific engagement project (I’m a Scientist (IAS), Get me out of here! or IAS Debate Kits) were invited via email to participate in Decipher my Data.[6,7] The email was sent to approximately 2,000 contacts representing 1,500 schools. Secondary schools in England with pupils aged 11–18 were eligible. We collected data for two influenza seasons in 2011/12 and 2012/13. Schools were all located in England, and, to make the results comparable to Public Health England’s national surveillance data, South, Central and North geographical regions were used to classify the region of each one taking part.[8,9]

After consent had been received from the head teacher, schools uploaded basic data including the number of pupils in each year group, the percentage of students in different ethnic groups, the percentage of children on free school meals and the full time equivalent number of teachers. Across all schools, absences were recorded as one of: medical (due to illness or medical appointment), authorized (for reasons other than illness or medical appointment) or unauthorised. Each week, schools were asked to submit the total number of half-day medical absences in each year group via the project website—http://flu.deciphermydata.org.uk/. Data on authorized and unauthorized absences were not included in the weekly submissions. Data submission was encouraged from week 38 (September) until week 13 and 12 (March) in the 2011/12 and 2012/13 seasons respectively. Data were collected for these periods as it was likely to capture the peak period of influenza circulation. A password protected website was built that enabled school teachers and pupils to submit data each week and take part in the learning activities specifically designed to engage students with data analysis. Detailed instructions were provided on how the data should be collected and uploaded, and telephone advice was provided in case difficulties were encountered. No face-to-face training was provided. Schools were asked to submit data in as timely a manner as possible, and email reminders about the submission of data were sent on an ad hoc basis.

No details were collected about the medical reason for each illness absence as schools do not routinely collect these. In the second year of the project, weekly school absence data were automatically time stamped when uploaded, allowing analysis of the time lag between the end of a school week and the time to submission on the project website.

The primary outcome was prevalence of daily school absence due to illness–referred to as school absence prevalence throughout the rest of this paper. This was calculated using the number of school absences per day as the numerator, and the number of pupils in years 7–11 (typically aged between 11 and 16) as the denominator. Consistent with previous studies, we analysed data for years 7–11 only, as children in years 12 and 13 (generally aged 16–18 years) tend to have higher levels of scheduled absence (due to the more variable nature of their school timetable and provision for personal study time) making the denominator data less reliable.[4] An additional reason for excluding years 12 and 13 was that all secondary schools taking part had years 7–11 but not all had years 12 and 13. Schools submitted the aggregate number of absences for each year group on a weekly basis, along with the number of half-day sessions at the school (e.g. 10 represented a full 5 day week).

Poisson regression was used to calculate daily school absence prevalence weighted by region. Weights were generated by using the proportion of children in years 7 to 11 for each region in England using school census data for 2010, and the proportion of children for each region taking part in this project and submitting their data each week.[10] Weighting was performed to account for differences in the proportion of children sampled in each region and proportion of all children attending schools that region. In weeks where there were no data submitted by schools for a particular region, weighting was calculated using the two remaining areas. We estimated daily school absence prevalence for each week and across the whole year. We also produced estimates for the first eight weeks of the study to examine levels outside of the main influenza season.

We anticipated the recruitment of around 100 schools. This would enable a 2% daily prevalence of school absence to be calculated with 95% CIs of 1.9–2.1% (off-peak influenza) and a 7% prevalence with 95% CIs of 6.8–7.2% (peak influenza).

We collected data from each school on a weekly basis which limited the number of data points available for analysis. Thus, it was not possible to conduct a more complex statistical investigation (such as a time series study) and we therefore took a descriptive approach to our analyses. Several descriptive analyses were performed to examine the number of schools submitting data each week, the time to upload data (i.e. lag between end of school week and receipt of data, second year of data only), the total number of pupils in the sample population and their characteristics and geographical distribution.

To examine the association between weekly weighted school absence prevalence and levels of influenza circulating in the community, results were triangulated graphically and through univariable linear regression against Royal College of General Practitioners (RCGP) influenza like illness (ILI) episode incidence rate per 100,000 population for all ages, and rates among individuals aged 5–14.[11] School absence data were also plotted against microbiological surveillance data (Respiratory Datamart) on the proportion of samples positive for influenza (A+B). Respiratory DataMart is based on laboratory results collated from a network of 16 PHE and NHS laboratories in England and includes respiratory swabs from primary and secondary care from people of all ages.[12] These swabs are tested for a variety of viruses using real time polymerase chain reaction assays.[8] All analyses were conducted for weeks 38 to 13, the periods when schools were being actively encouraged to submit their data. Final end of year reconciled RCGP and Respiratory Datamart data were supplied by Public Health England and University of Surrey (who maintain the RCGP system). Norovirus data were taken from Public Health England’s weekly health protection report.[13]

School absence prevalence was plotted against Respiratory Datamart data for respiratory syncytial virus (RSV) and Rhinovirus and laboratory confirmed cases of Norovirus.[13] These infections are common in children and might explain trends in school absence prevalence. This counterfactual analysis was therefore conducted to examine this possible alternative explanation for any associations found by triangulating results graphically and through univariable linear regression. All analyses were conducted in Stata v.13 (Statacorp LP, College Station, TX, USA).

This work was conducted as a public engagement in science project and several interactive lesson plans were developed for schools taking part. Topics covered in these sessions included an introduction to the data, how to analyse the results, and how to write up the results. The project team wrote regular blogs that were posted on the study website and emailed to students and teachers. During the first year of the project, students were able to post questions to scientists taking part, and in both years of the project, students were able to write ‘Lab logs’ about their observations and analysis, to which the authors responded. As this project recruited schools rather than individual children, written informed consent was received from the head teacher of each school taking part—as approved by UCL research ethics committee under ethics application number: 3294/001.

## Results

### Establishing an electronic system for the collection of routine school absence data

Following an introductory email in the summer term of 2011, 352 teachers expressed an interest in taking part, representing approximately 250 schools. At the end of 2011/12 season 31 schools were registered on the site. By the end of the 2012/13 there were a total of 89 schools registered. Across the two seasons, a total of 47 schools returned consent forms and 27 submitted data at least once. There were a greater number of schools in PHE’s southern (16/27) compared to central (8/27) and northern geographical regions (3/27).

**Fig 1:**
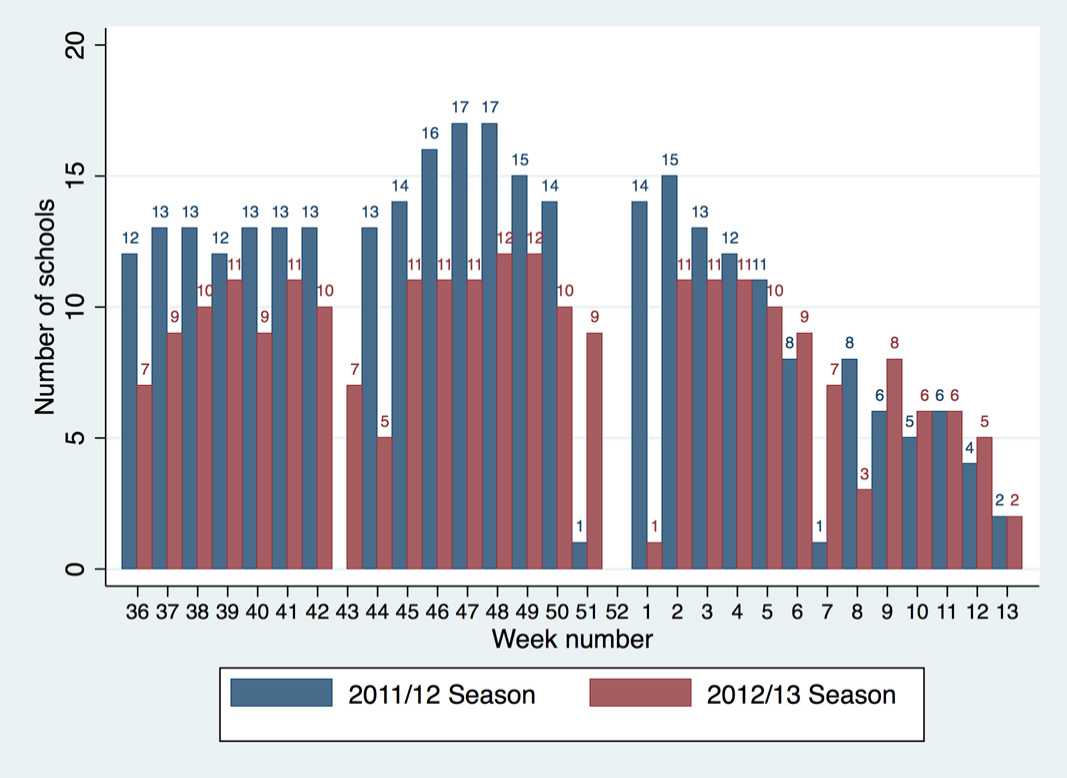
Number of schools submitting absence data each week.

Schools were encouraged to submit absence levels from week 38 until week 13 in the 2011/12 season and until week 12 in 2012/13 (the start of Easter holidays). Several schools provided historical data from week 36 and continued to submit data until week 27 in 2011/12 and week 19 in 2012/13. A mean of 11 (range: 0–17; includes school holidays hence lower end of range is zero) schools submitted data each week during school terms of the 2011/12 season and 8 schools (range: 0–12) during 2012/13 (Fig 1). Data from these schools provided absence information for a mean of 10,231 pupils per week in the 2011/12 and 7,743 pupils per week in 2012/13. No data were uploaded in weeks 43 and 7 in 2011/12, as these were school half terms. In both years no data were uploaded for two weeks during the Christmas holidays. As there was variation in the timings of half term across the schools taking part during the 2012/13 season, data were uploaded for all other weeks.

### Determining if school absence prevalence is associated with established surveillance measures of influenza

**Fig 2:**
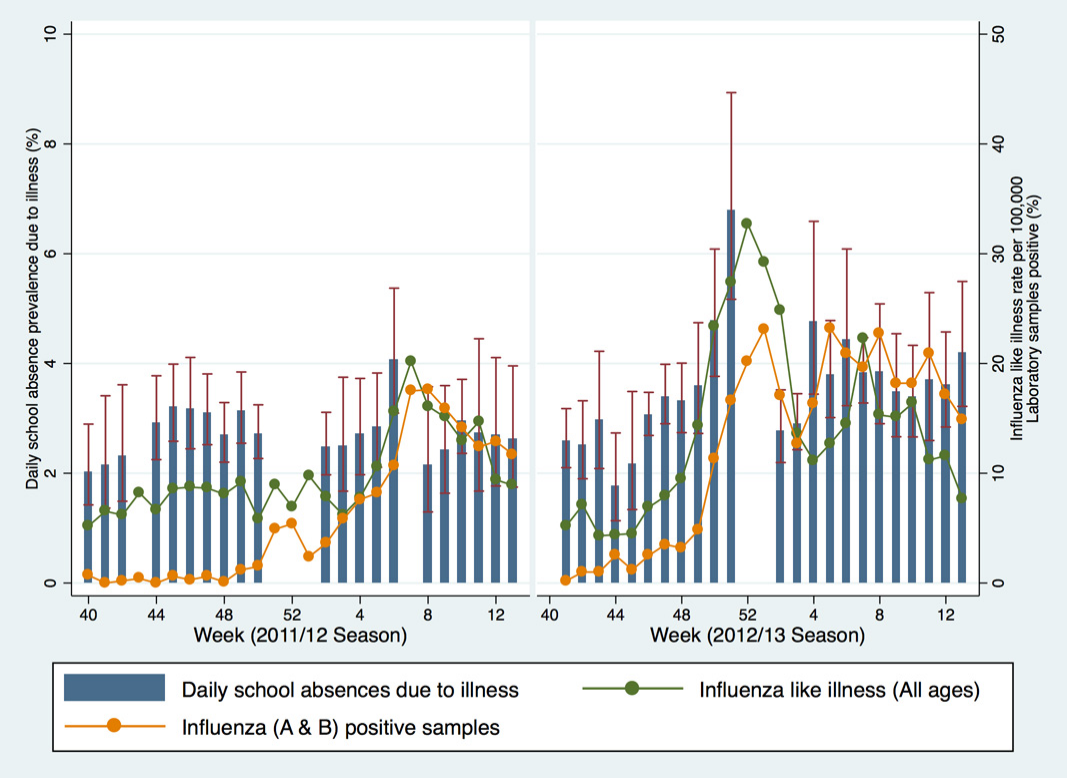
Daily school absence prevalence (years 7–11), weekly influenza like illness consultation rates to sentinel general practices in England (all ages), and weekly proportion of samples positive for influenza A & B (all ages) from Respiratory Datamart. Fig 2 note: data underlying this graph are available at UCL Discovery.[14]

During 2011/12, the mean weighted daily prevalence of school illness absence for children in years 7 to 11 was 2.7% (95% CI 2.5, 2.9) between week 38 and 13. During the first eight weeks of the season the mean weighted daily prevalence of school illness absence for children in years 7 to 11 was 2.6% (95% CI 2,4, 2.7). There was a peak in the mean weighted daily prevalence of school illness absence of 4.1% (95%CI: 3.1, 5.4) in week 6 (Fig 2). Week 7 was a school half term and therefore no data were submitted, and levels in week 8 reduced back to 2.2% (95%CI: 1.3, 3.6). RCGP ILI data (all ages) started to increase in week 5 and peaked during week 7 at 17.2 per 100,000 during the 2011/12 season. This peak was later than seen in previous years and did not cross the pre-epidemic threshold at that time of 30 per 100,000 for the entire season, indicating low influenza activity. Respiratory Datamart microbiological surveillance data on the proportion of samples positive for influenza (A+B) peaked in week 9 at 22.2%, which was lower and later than the previous two seasons.[8]

During 2012/13, the mean weighted daily school absence prevalence was 3.4% (95% CI 3.1, 3.7) between week 38 and 12. During the first eight weeks of the season the mean weighted daily prevalence of school illness absence for children in years 7 to 11 was 3.3% (95% CI 3.0, 3.5). School absence prevalence peaked in week 51 at 6.8% (95%CI 5.2, 8.9). School absence prevalence reduced to mean seasonal levels in week two at 2.8% (95%CI 2.2, 3.5). RCGP clinical surveillance data of ILI (all ages) peaked at 32.3 per 100,000 in week 1. The percentage of samples positive for influenza (A+B) in Respiratory Datamart was highest in week eight (26.4%) but also peaked in week 52 at 25.7%. (10) Schools data generally peaked one or two weeks before the national surveillance data.

**Fig 3:**
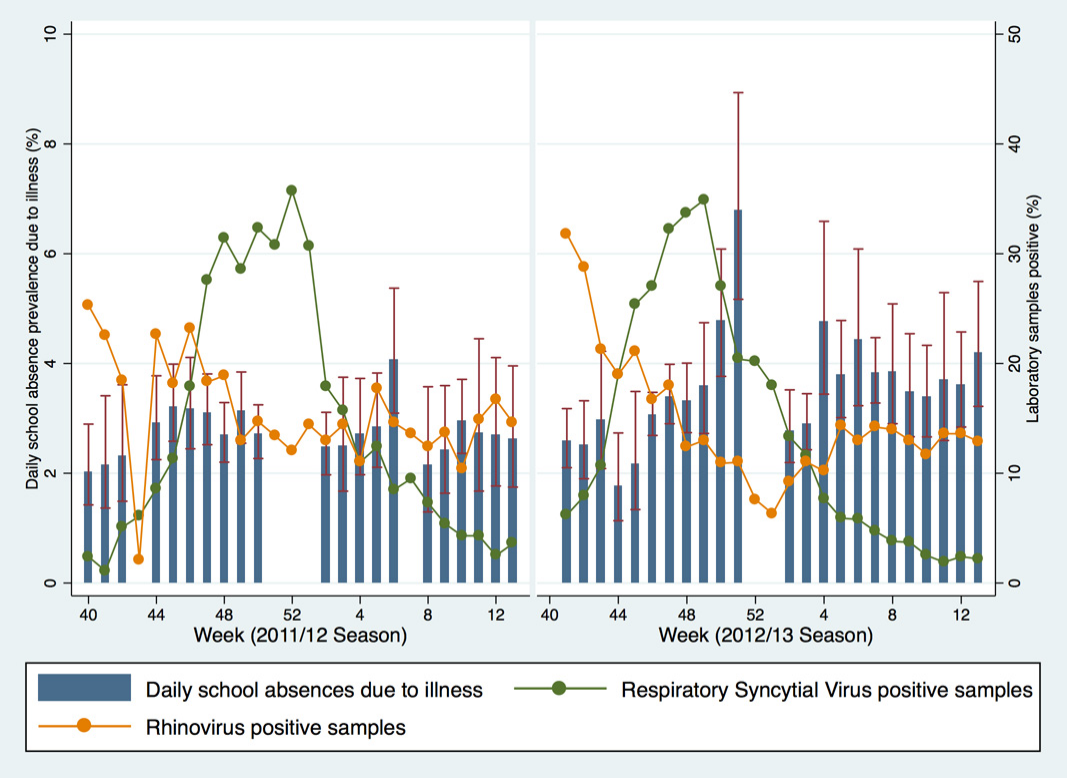
Daily school absence prevalence (years 7–11) and the weekly proportion of samples positive for RSV and Rhinovirus from Respiratory Datamart. Fig 3 Note: Rhinovirus data for weeks 40 (69%) and 41 (55%) in 2011/12 season plotted at 50% to preserve axis scales. Data underlying this graph are available at UCL Discovery.[14]

**Fig 4:**
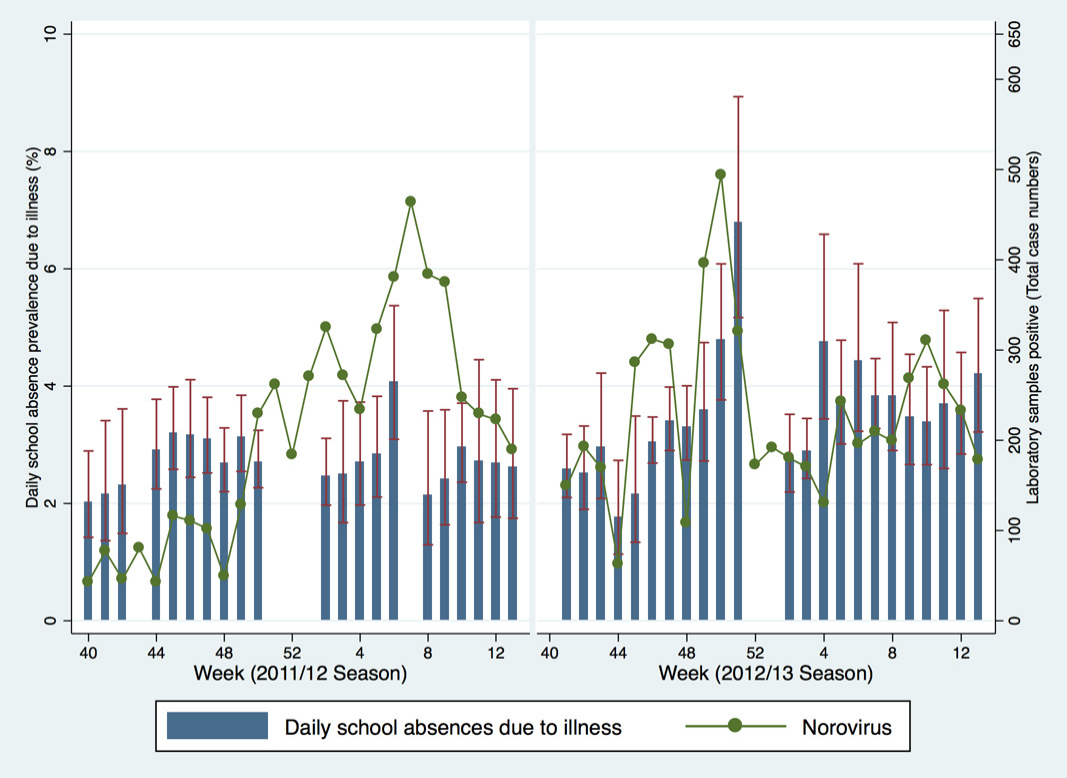
Daily school absence prevalence (years 7–11) and the weekly number of laboratory confirmed cases of Norovirus. Fig 4 Note: Data underlying this graph are available at UCL Discovery.[14]

Microbiological surveillance data for RSV in 2011/12 showed the highest percentage of positive cases in week 52 at 33.6% (Fig 3). In 2012/13 the peak of RSV occurred in week 49 at 33.1%. The percentage of samples positive for Rhinovirus was highest during week 40 at 69%. Rhinovirus levels were also highest at the beginning of the 2012/13 season (30.5% in week 41). The total number of virologically confirmed cases of Norovirus peaked at 464 in week 7 during 2011/12 and 494 in week 50 in 2012/13 (Fig 4). There was no obvious descriptive association between school absence prevalence and these non-influenza respiratory and gastrointestinal infections across the two seasons.

There was evidence for a positive linear association between school absence prevalence across the two seasons with RCGP ILI data for all age groups. For every 1% increase in school absence prevalence, the rate of ILI went up by 4.5 per 100,000 (95%CIs: 2.9, 6.1; p-value <0.001; R^2^ 0.42; Table 1). There was also strong evidence for an association with RCGP influenza like illness in children aged 5–14 years. For every 1% increase in school absence prevalence, the rate of ILI in those aged 5–14 years went up by 8.9 (95%CIs: 6.8, 11.0; p-value <0.001; R^2^ 0.62). The proportion of samples positive for influenza A + B submitted to Respiratory Datamart increased by 5.2 for every 1% increase in school absence prevalence (95%CIs 2.6, 7.9; p-value <0.001, R^2^ 0.27). The proportion of variance explained by linear regression was greatest for RCGP ILI in children aged 5–14 years, which explained 62% of the association. There was some evidence for a linear association with Rhinovirus (negatively correlated) and Norovirus (Table 1).

**Table 1:** Univariable linear regression models examining association between weighted school absence prevalence (years 7–11) and surveillance data for respiratory infections and Norovirus.

Surveillance data	Beta coefficient (95% CIs)	R^2^	p-value
RCGP influenza like illness (All ages, rate per 100,000)	4.5 (2.9, 6.1)	0.42	<0.001
RCGP influenza like illness (5–14 years, rate per 100,000)	8.9 (6.8, 11.0)	0.62	<0.001
Influenza A + B (Percentage of laboratory samples testing positive)	5.2 (2.6, 7.9)	0.27	<0.001
RSV (Percentage of laboratory samples testing positive)	0.5 (-2.9,)	0.002	0.78
Rhinovirus (Percentage of laboratory samples testing positive)	—5.5 (-8.7, -2.0)	0.19	0.002
Norovirus (Total number of laboratory samples testing positive)	38.3 (3.0, 73.6)	0.10	0.034

## Public Engagement in Science

32 Lablogs were completed in year 1 and 18 in year 2 from four schools in total. Year 1 also had 50 questions from students that were answered by scientists. The project website was visited 13,604 times by 8,266 unique visitors over the project period and 46,750 pages were viewed. Website activity was slightly busier in year one. Full results of an independent evaluation of the public engagement aspect of the first year of Decipher my Data are available online.[7] Schools taking part in the project were able to provide interesting additional insights and potential explanations for differences between data from their school and national school absence prevalence, such as weather, teaching environment and other characteristics about the school or local area.

## Discussion

This study aimed to establish an electronic system for influenza surveillance using public engagement with science, and investigate whether school absence prevalence was correlated with established surveillance measures for influenza. The project ran during the 2011/12 and 2012/13 seasons and recruited 47 schools with data submitted by 27. Linear regression showed an association between school absence prevalence and RCGP reported ILI, laboratory confirmed cases of influenza A & B, and some evidence for a linear association with Rhinovirus (negatively correlated) and Norovirus.

### Strengths and weaknesses of the study

This study has demonstrated the feasibility of establishing a novel mechanism for influenza surveillance in schools using public engagement in science. The study not only resulted in academically interesting findings but also served an important educational purpose. Students taking part were provided with a unique opportunity to analyse data and learn about basic epidemiological concepts and the interpretation of scientific results. The fact that levels of influenza were low during the first year, whilst frustrating from a research perspective, was educationally useful, demonstrating how not everything in science goes exactly according to plan.

There are several limitations of using school absence data for surveillance of influenza activity in the community, particularly the fact that it is not possible to collect any data during school holidays or at weekends. Whilst only medical absence data were collected, the clinical reasons for the illness absence were not collected, and despite using only data for medical absences it is likely that a very small number may have been misclassified e.g. a medical absence that was not actually due to an illness or medical appointment.

Our analyses are descriptive and rely on graphical and bivariate regression. It is therefore not possible to rule out potential confounding factors as alternative explanations for our findings. It is also only possible to provide associations between exposures and outcomes in our analyses rather than more sophisticated and adjusted measures of effect. We were not able to look at potential behavioural issues that may have affected the results of our study. For example, it is possible that public awareness of an increase in cases of influenza-like illnesses in the community (for example through media coverage) may influence the likelihood of parents removing their children at an earlier stage from school when they observe in them the possible symptoms of influenza. This could potentially impact on the timings of peaks in school and surveillance data in our study. In both seasons, our descriptive analysis of the data suggests that a peak in school absence prevalence data occurred at a minimum of one week before national surveillance data. Due to the limitations of our data and the analytical methods we were able to apply to it, we have not been able to provide stronger statistical evidence for a lag between school absence and RCGP ILI or laboratory confirmed cases of influenza A & B.

Schools taking part in Decipher my Data were predominantly based in the south of England, and despite weighting results to make them nationally representative, the convenience sampling and low number of schools in the north of the country may have led to a bias and greater levels of uncertainty within these estimates. Schools were given detailed instructions on how to collect and upload data, however, measurement bias (due to inconsistencies in the way the data were processed and uploaded may have varied across schools taking part) is likely to have been randomly distributed and will therefore lead to a non-differential bias and a reduction in the study power.

### Comparison to existing literature

A previous study conducted in England recruited eight primary schools and three secondary schools during the 2005/6 season.[4] This study was carried out for one season and included self-reported cause of the illness by the parent or guardian at the time of notifying the school about a child’s absence. The RCGP ILI peak occurred one week after school absence data. A similar study was also performed during 2005–2007 using results from six primary schools in a single local authority in East London.[5] This study was able to calculate both the incidence and prevalence of school absences from the data collected. Peaks in the prevalence of school absence showed a greater correlation with laboratory confirmed cases of influenza A & B than incidence rates.

A further study used public engagement in science to investigate contact patterns relevant to transmission of influenza and other direct transmission infections in young children.[15] The study used children aged 13–15 to capture mixing patterns in children aged 4–11. Data collection questionnaires were designed in association with the school children and administered by the students aged 13–15. The study found evidence of sex-specific assortive mixing within and between classes in the same school, and a marked social structure. The authors concluded that the methods were a helpful way to examine mixing patterns in this difficult to research group.

### Interpretation of the findings

The results of this study provide evidence that school absence prevalence could be a useful tool for surveillance of influenza in children aged 11 to 16 and may have utility for providing earlier warning of an outbreak than existing measures. The data are likely to be more representative of the community burden of disease in children compared to existing surveillance data. Schools were actively engaged in the collection and analysis of the data. It was not possible to establish with certainty whether school absence prevalence detected outbreaks of disease earlier than existing data, or whether peaks occurred at an earlier stage, and future work should be carried out to examine these possibilities as such data might enable a more timely public health response to influenza epidemics and pandemics. The network of web-based data collection platforms we established in schools through active engagement provides an innovative model of conducting scientific research and could be used to answer a range of other study questions (for both influenza and other infections) in the future.
